# Chloralamid

**Published:** 1890-04

**Authors:** Chas. H. Steele

**Affiliations:** Professor of Materia Medica and Therapeutics, Cooper Medical College.


ARTICLE III.

### CHLORALAMID.

BY CHAS. H. STEELE, A.M., M.D.

[Professor of Materia Medica and Therapeutics, Cooper Medical College.]

From *Pacific Medical Journal*

Chloralamid is properly a chloralformamid or formidate of chloral with the formula $CCL3CH \left\{ \begin{matrix} OH \\ NHCHO. \end{matrix} \right.$

It is chemically a union of chloral anhydride (CCI2-CHO) with formamide (CHO . NH2).

Prof. J. von Mering was its discoverer and E. Schering, of Berlin, its exclusive manufacturer. It occurs in the form of colorless, very faintly but not unpleasantly bitter, non caustic, odorless crystals, melting at 239° F. and soluble in from nine to fourteen parts of cold and less of warm water, in one part of absolute and one and a half parts of ninety-six per cent. alcohol. To understand its time of action it is well to remember that it requires five hours to dissolve twenty grains in two ounces of water and only fifteen minutes when the menstrum is one dram of rectified spirit. No precipitation occurs on adding the alcoholic solution to water.

TESTS.

Upon heating chloralamid to its melting point, 239° F., chloral is liberated and may be tested separately. Heated with a solution of potassa it emits odors of chloroform and ammonia. A few grains in a solution of four drops of ninety per cent. carbolic acid in one-half ounce of strong sulphuric acid gradually heated to boiling gives rise to a bright-red color and a strong odor of chlorine. With the same test phenacetin produces a dark purplish-brown mixture with a strong acetous odor and sulphonal, a bright green, changing to dark green upon the further addition of strong sulphurous acid. Fehling's and Pavy's solutions are not affected by chloralamid.

INCOMPATIBLES.

The drug is rapidly decomposed by water heated above 140° F. and by caustic alkalies, and slowly by alkaline carbonates.

ADMINISTRATION.

The dose varies from fifteen to sixty grains, while the majority of experimenters rely upon a single dose of thirty grains and rarely find forty-five grains necessary. A few consider this last dose as the limit of safety. A child of eleven years of age was given seven grains, and another four and a half years old, five and ten grains. In any case it is better, especially with new drugs, to commence with the smallest dose and increase cautiously. To secure the best results the drug should be administered from one to one and one half hours before bed-time. It may be prescribed in powders alone or triturated with oleosacchara fœniculi, capsules, wafers or dissolved in wine or brandy, to which water may be added as desired. Some difficulty will be experienced in taking the powder in water, tea or milk, as advised by a few, on account of its slow solution and tendency to adhere to the sides of the vessel. It is better to wash down the powder with a draught of milk, weak tea or water. An advised prescription is :

| | | | |
|---|---|---|---|
| R | Chloralamid - - | gr. xiv. |
| | Acidi Hydrochlorici Diluti - | gtt. vi. |
| | Syrupi Rubi Idæi - - | ʒ ii. |
| | Aquæ q. s. ad. - - | ℥ ii. |

Sig. To be taken in one or two doses.

As an enema, in which form it is unirritating and slow in action, we may use :

| | | |
|---|---|---|
| R | Chloralamid - - | gr. xiv. |
| | Acidi Hydrochlorici Diluti - | gtt. iii. |
| | Alcohol - - - | *m* xx. |
| | Aquæ - - - | ℥ iii. |

So administered it is considered by a few to be most reliable in its action.

However used it must be remembered that its solution is not to be heated.

PHYSIOLOGICAL ACTION.

Locally, chloralamid has been found to be absolutely free from irritation, and even where a ten per cent. solution has been applied to the delicate conjunctiva. Internally, no effect has yet been discovered upon digestion and circulation, except in relation to the vaso motor centre. Here we have a trivial difference in opinion. A single authority (Langgaard)—a possible pessimist, for he also makes the same statement regarding the respiratory centre—affirms that blood pressure is lowered through depression of the vaso moator apparatus. Reichmann admits that this is slightly so, but von Mering, Zuntz, Prof. Leech of Guy's Hospital, Geo. P. Cope of Dublin and many others deny this action, while Rabow, of Lausanne, goes still further and states that the formamide, liberated from the chloral, stimulates the vaso motor centre in the medulla and raises blood pressure.

Chloralamid has the property, of inducing an apparently natural sleep, commencing in from one-half to three hours and lasting from six to ten hours. The usual interval between the administration of the dose and the advent of sleep is from one to two hours, but this depends so greatly upon the slow solubility of the drug in the watery fluids of the stomach that it is possible that absorption may not be completed or sleep commence until the morning after the evening dose. This delay might be obviated by employing an alcoholic solution. In some cases the sleep is interrupted and even many failures are reported. However, all this depends upon the dose administered, forty-five grains, equalling thirty grains of chloral hydrate, being considered necessary to insure certainty. The frequency of the failures may be estimated from the fact that Dr. Cope reports only four per cent., Dr. Williams six and one-half per cent. of all cases, and Prof. Leech no failures in nineteen patients. Some consider the sleep to be deeper than that obtained with chloral.

To what this hypnotic action is to be attributed is, as yet, only a matter of conjecture. Dr. Eugen Kny supposes that, in the alkaline blood, chloral is gradually liberated, and he partially bases this opinion upon the presence of uro-chloralic acid in the urine. It would seem, however, that more depression would have been discovered if such were the case. The only depression positively established was that of reflex action in frogs after injecting one-third to one-half of a grain.

### INCIDENTAL EFFECTS.

While no unpleasant after-effects were noticed by many authorities, no disturbance of the heart, respiration, temperature, kidneys, digestion, or appetite, (but rather improvement of appetite, according to Dr. D. R. Paterson), a few have occasionally found slight headache upon awakening, alone or with lassitude and a desire to sleep during the next morning or entire day. Among the other unusual effects are thus arranged in order, commencing with those most frequently reported: slight or severe vertigo, thirst, nausea, dryness of the mouth, loss of appetite, slight delirium, vomiting, cardiac weakness, rapid and feeble pulse and restlessness which necessitated forcible restraint. The more severe symptoms appeared after large doses, over thirty grains, and were not consecutive or persistent as is the case with sulphonal. Patients do not seem to become accustomed to its use, nor is there evidence that the drug is cumulative in action.

One hour after a dose of sixty grains, there have, in two instances, appeared vertigo, intoxication, volubility, inco-ordination, occipital headache, nausea and either no change or slight increase in the pulse rate. These symptoms were at their height in about three hours after the dose, while slight vertigo and cephalalgia persisted during the second day.

These incidental effects seem to be very rarely exhibited, even after the largest therapeutic dose, and are proportionally not more frequent than with chloral or morphine.

THERAPEUTICS.

Chloralamid is successfully employed in conquering insomnia, and particularly that form denominated simple or ideopathic insomnia, not due to excitement or severe pain. It is; furthermore, possible for the wakeful patient to enjoy several nights of natural sleep after a single dose. The best results occur when the drug is used in insomnia due to nervousness, neurasthenia, hysteria, "spinal disease" or old age; next best when the causes are chronic alcoholism, alcohol excess, cardiac and bronchial asthma, pleuritis, phthisis, pericarditis, arterial sclerosis, organic heart disease, typhoid fever, gastritis, subacute nephritis, ascites, diabetes, mellitus and in the morphine habit. It is less effective when wakefulness is due to tabes dorsalis, neuralgia, progressive paralysis, the excitement of insanity, cerebral softening with delirium, melancholia, chronic mania and acute mania. In these conditions, doses of from thirty to sixty grains are required, providing such doses are tolerated.

The drug is useless when the insomnia results from paralytic dementia, maniacal excitement or hallucinations, severe neuralgia or other pain, violent cough, distressing headache, delirium of cerebral apoplexy and from delirium tremens.

Even pain, when not acute, is often relieved, and the large doses necessitated are, by many patients, preferred to morphine. Chloralamid, in doses of from twenty to sixty grains, has checked the pains of thoracic aneurism, carcinoma of the stomach and liver, sarcoma of a rib, erysipelas, rheumatic fever, floating kidney, neuralgia, gallstone, varicose ulcer and alcoholic neuris.

In Chorea, a boy of eleven years of age was cured in five days by fifteen grains of the drug three times daily, and in like manner, a girl, after receiving no benefit from other forms of treatment, was afforded relief in eight days.

When administered in phthisis it was found that the troublesome night sweats disappeared.—*Notes on New Remedies.*

